# Children's eating attitudes test (ChEAT): reliability and validation in German children and adolescents based on clinical data

**DOI:** 10.1007/s40519-025-01773-w

**Published:** 2025-08-14

**Authors:** Lena Nonnast, Laura Maria Derks, Natalie Deux, Martin Holtmann, Tanja Legenbauer

**Affiliations:** LWL University Hospital Hamm for Child and Adolescent Psychiatry, Psychotherapy and Psychosomatic, Ruhr-University Bochum, Hamm, Germany

**Keywords:** Children's eating attitudes test, ChEAT, Eating disorders, Psychometric characteristics, German children and adolescents

## Abstract

**Purpose:**

The objective of this study is to assess the validity of the German version of the Children's Eating Attitudes Test (ChEAT), an internationally used tool for the detection of eating disorder (ED) symptoms, in a clinical sample.

**Methods:**

The ChEAT self-report questionnaire, comprising 26 items, was employed to examine eating behaviors of a clinical sample of 342 adolescents (aged 12–18 years) undergoing inpatient treatment at a child and adolescent psychiatric clinic in Germany. The ChEAT was validated through an exploratory factor analysis (EFA), followed by a confirmatory factor analysis (CFA) and an examination of internal consistency. Subsequent analyses were conducted to identify differences associated with participant characteristics, including age, gender, body mass index (BMI), and diagnosis. Furthermore, additional eating behaviors, depression, and anxiety symptoms were documented via supplementary questionnaires and correlated to the ChEAT to evaluate the convergent and discriminant validity.

**Results:**

The factorial validity of the ChEAT was confirmed through EFA and CFA, resulting in a five-factor structure with the following dimensions: 'Body and Weight Concern,' 'Dieting,' 'Social Pressure,' 'Purging and Binge Eating,' and 'Food Preoccupation'. The 24-item model showed high internal consistency and demonstrated an acceptable fit to the data. Convergent and discriminant validity of the ChEAT was supported by significant correlations with other self-report questionnaires. Higher ChEAT average scores were observed in females and those with a history of eating or depressive disorders, whereas age or BMI showed no correlation.

**Conclusion:**

The data demonstrate that the German version of the ChEAT appears to be a reliable and valid instrument for identifying ED symptoms in clinical samples. However, further studies are necessary to evaluate the factor structure and validity.

**Level of evidence:**

V, cross-sectional, descriptive study.

**Supplementary Information:**

The online version contains supplementary material available at 10.1007/s40519-025-01773-w.

## Introduction

Eating disorders represent a group of clinically distinct conditions, including anorexia nervosa (AN), bulimia nervosa (BN), and binge eating disorder (BED), which may frequently manifest with overlapping symptoms [1]. These disorders are characterized by disturbed eating patterns and an excessive preoccupation with food. A core symptom of AN is excessive weight loss, typically achieved through restrictive dieting and/or active weight loss measures such as excessive exercise or laxative abuse. BN is characterized by repeated binge eating episodes accompanied by a severe loss of control, followed by compensatory behaviors such as vomiting to prevent weight gain. In contrast, BED involves regular binge eating without compensatory behaviors. Both BN and BED often co-occur with overweight or obesity [2–4].

Individuals of all ages are susceptible to eating disorders, which have a global prevalence. In Europe, the prevalence rates for AN are 0.5–2%, for BN are 1–3%, and for other eating disorders are 4.8%. The highest incidence of eating disorders occurs between the ages of 13 and 18 [5, 6]. A 2019 study on the health of children and adolescents in Germany revealed that 19.8% of individuals between the ages of 11 and 17 exhibited symptoms associated with eating disorders [7]. Recent data indicate an increase in the prevalence of eating disorders in Germany following the onset of the COVID-19 pandemic. This is evidenced by a 20% rise in the administrative prevalence in the outpatient sector among girls, as well as a surge in inpatient admissions, from 10.5 to 16.7% [8, 9]. The risk is significantly higher for girls and young women, with a tenfold increase in the likelihood of developing eating disorders [10–12]. Psychological comorbidities are prevalent in individuals with eating disorders, with an overall comorbidity occurring in over 70% of patients. Depressive disorders appear to play a special role in this context, as some studies have identified them as the most common comorbidity. In some cases, depression appears to be exacerbated or manifested by eating disorder symptoms. Other common comorbid disorders are anxiety disorders and alcohol and drug abuse [13–17].

Research in psychopathology increasingly focuses on eating behaviors and body attitudes in children and adolescents, which are considered key factors in the development and maintenance of eating disorders [18, 19]. Early recognition and intervention are essential, given the severe physical and psychological consequences of eating disorders [20]. Long-term follow-up studies indicate that individuals may experience persistent symptoms and illness-related restrictions, such as unemployment and comorbid mental health issues, requiring psychotherapy [21]. Additionally, AN has the highest mortality rate among mental illnesses, with an average of 5%, depending on the study [22–24]. The development of culturally and linguistically appropriate tools is of paramount importance for the identification of early signs of disordered eating behavior in children.

One such tool is the Children's Eating Attitudes Test (ChEAT), which is commonly employed to evaluate eating behavior and attitudes in children aged 8 years and older. This self-report questionnaire is widely used in international cohort studies and is considered a valid instrument, with Cronbach's alpha values ranging from .71 to .88 [25, 26]. The ChEAT has been translated into a variety of languages and evaluated, including Spanish, French, Japanese, Chinese, Russian, Finnish, Portuguese, Dutch, and Turkish, among others [25–34]. The participants in the validation studies were predominantly schoolchildren between the ages of 6 and 18 years, although some studies have also evaluated the ChEAT in clinical samples, including patients with eating disorders or obesity [26, 28, 35]. Psychometric analyses of linguistically adapted versions have demonstrated significant variations in the number of factors and the allocation of items. These differences are likely explained by cultural variations in symptoms and language. While the original English version of the ChEAT demonstrated a four-factor structure, subsequent studies have identified between three (e.g. Turkey) and seven (e.g. Poland) factors [33, 36]. In terms of content, the factors deal with dieting attitudes, food preoccupation, body concerns, and fear of gaining weight. Some studies also include factors related to purging and restrictive behavior. With regard to the number of items used, short versions with only 12 items have now been tested in Canada, for example, whereas other studies continue to use between 24 and 26 items of the original version [29, 31, 34]. Convergent and discriminant validity of the ChEAT have been confirmed by several studies, which have also correlated the ChEAT for example with the Three-Factor Eating Questionnaire and the Children's Depression Inventory [26, 35] (for additional information regarding the international study situation, refer to Table 1 in the supplementary information). This study aims to assess the reliability and validity of the German version of the ChEAT in a clinical sample of 12 to 18-year-olds, focusing on psychometric properties, factor structure, and both convergent and discriminant validity, based on correlations with self-report questionnaires on eating disorders, depression, and anxiety. We chose not to employ a priori hypotheses due to the significant discrepancies observed in prior validation studies.

**Table 1:** Characteristics of participants

Baseline characteristic	*n*	%
*Gender*		
Girls	197	57.6
Boys	145	42.2
*Age*		
12	20	5.8
13	50	14.6
14	49	14.3
15	72	21.1
16	68	19.9
17	74	21.6
18	9	2.6
*BMI percentiles*^*a*^		
Underweight (P3- < P10)	31	9.1
Normal weight (≥ P10- ≤ P90)	172	50.3
Overweight (> P90- ≤ P97)	34	9.9
Obesity (> P97)	57	16.7
Incomplete data	48	14.0
*Main diagnosis*^*b*^		
Depressive disorder	129	37.7
Mixed disorders of conduct and emotions	106	31.0
Mental and behavioral disorders due to use of cannabinoids	28	8.2
Emotional disorders with onset specific to childhood	16	4.7
Phobic anxiety disorders	11	3.2
Reaction to severe stress, and adjustment disorders	10	2.9
Anorexia nervosa	9	2.6
Others	33	9.7
*Secondary diagnosis of an eating disorder*^*b*^		
Anorexia nervosa	2	0.6
Atypical anorexia nervosa	2	0.6
Bulimia nervosa	2	0.6
Overeating associated with other psychological disturbances	1	0.3
Eating disorder, unspecified	1	0.3
*Comorbidity*		
No	131	38.3
Yes	211	51.7
*Medication*^*c*^		
Yes	125	36.5
No	217	63.5

*N* = 342; BMI = Body mass index; ^a^BMI-percentiles were based on the norms established by Kromeyer-Hauschild [37]; ^b^Diagnoses were based on the International Classification of Diseases, 10th Revision (ICD-10, World Health Organization [38]), in accordance with the standard guidelines of the German healthcare system; ^c^Medication in context of psychotropic drugs

## Methods

### Participants

Participants were recruited during inpatient treatment at the LWL University Clinic Hamm of the Ruhr-University Bochum for Child and Adolescent Psychiatry, Psychotherapy, and Psychosomatics in Germany. The clinic is one of the largest psychiatric institutions in Germany, with a treatment capacity of 110 inpatients, 68 day care patients, and outpatients. It caters to children and adolescents aged 5–18 years from a drainage area of 1.5 million people residing in smaller cities (with a population of 100.000 or more) and surrounding areas. The clinic treats up to 1300 patients per year with significant functional impairments. The spectrum of diagnoses ranges from depression to school avoidance, obsessive–compulsive disorders and psychotic illnesses.

All study participants were undergoing inpatient treatment at the time of recruitment. Admission was determined by the presence of severe functional impairments, particularly in the emotional or social aspects, with the primary diagnosis serving as the basis for treatment allocation. However, only patients who are able to complete the tests and are in the hospital for at least 4 days take part in the routine diagnostics, i.e. patients with acute suicidal tendencies, psychotic symptoms etc. are not included. Participants were recruited as part of a larger study on the relationship between reduced sleep and weight status in children and adolescents with affective dysregulation. Participants were included in the study on a consecutive basis from 2013 to 2014. Participants who participated at the routine diagnostic assessment at the beginning of their inpatient treatment were asked whether they wanted to participate at an additional diagnostic assessment dealing with impulsivity and eating behavior. Those who consented to participate were informed about the study's content, and both the participants and their legal guardians provided informed consent. Subsequently, a second assessment time point was established, and the participants underwent a questionnaire-based test battery and underwent a measurement of their weight and height. As this study constitutes a secondary data analysis of the questionnaire-based data sets, only these will be described here. Additional information, such as sociodemographic data, anthropometric data, and the participants' diagnoses, was obtained from the electronic medical record. The study was granted ethical approval by the local ethics committee (no. 4854-13).

The sample consisted of 342 participants, with slightly more females than males and a mean age of 15.10 years (SD = 1.60). Approximately half of the participants were of normal weight with an average BMI of 23.29 kg/m^2^ (SD = 5.71). The average BMI was in the 65th percentile (SD = 32.55). The range of BMI values was between 12.7 and 49.2 kg/m^2^, corresponding to the 0th and 100th percentiles.

The most common main diagnoses were affective disorders and behavioral and emotional disorders with onset in childhood and adolescence. In total, approximately 5% of participants had an eating disorder as a primary or secondary diagnosis. Over half of all participants had one or more comorbidities, with a maximum of four secondary diagnoses at the time of recruitment (*M* = 1.88, SD = 0.86). Furthermore, just over a third of participants were taking medication. Table 1 provides an overview of the characteristics of all participants.

### Measures

#### Children’s eating attitudes test (ChEAT)

The ChEAT is a language and age-adapted version of the Eating Attitude Test for children aged 8 years and older [39, 40]. The questionnaire comprises 26 items that cover a wide range of attitudes and eating behaviors. Respondents answer the questionnaire on a 6-point Likert scale ranging from 1 'never' to 6 'almost always'. To calculate the total score, zero points are given to the first three answer levels (never, rarely, sometimes), and one to three points to levels four through six (often, very often, always). Items 19 and 25 are inversely coded items and should be scored accordingly. The maximum total score is 78 points, with higher scores indicating greater symptom severity. Previous studies have investigated different cut-off values, but an optimal cut-off value has not yet been agreed upon [25, 27, 28]. The ChEAT has demonstrated sufficient reliability and internal consistency in large international cohort studies [27, 28, 31, 33]. The original English version underwent psychometric analysis, revealing a four-factor structure with the subscales 'Dieting', 'Restricting and Purging', 'Food preoccupation', and 'Oral control' [18].

The research group at the LWL University Clinic Hamm translated the original English version into German. A native speaker then retranslated it, and any deviations were discussed and adapted with the advice of the native speaker.

#### Food cravings questionnaire—reduced (FCQ-T-r)

The FCQ-T-r is a modified German version of the FCQ, which is the most frequently used questionnaire for measuring food cravings [41, 42]. It consists of 15 items that use a 6-point Likert scale to measure behavioral, cognitive, and physiological aspects of food consumption. To the best of our knowledge, there are currently no published data on the use of the test in adolescent samples. The sample of Meule et al. had a mean age of 24 years [41]. The internal consistency of the current sample was high, with a Cronbach's alpha value of .93, which is consistent with previous studies [41].

#### Three-factor eating questionnaire (TFEQ)

The German version of the Three-Factor Eating Questionnaire comprises 51 items that measure dimensions of eating behavior on three subscales: 'Cognitive restraint of eating', 'disinhibition', and 'hunger' [43, 44]. Only the 'disinhibition' subscale, which includes 16 items, was used. This is because the study in which the data were originally collected examined interactions between increased weight and impulse control. The 'disinhibition' subscale seemed the most appropriate for this purpose. In Germany, the test is utilized for children and adolescents. Unfortunately, the original German version was not available for further statistical indices to be obtained. In the original publication by Stunkard et al. , a sample of people aged 17 to 77 was used, which at least indicates possible use in adolescence [44]. Psychometric studies have also reported acceptable reliability and validity. Cronbach's alpha was .74 for this sample.

#### Beck depression inventory (BDI-II)

The severity of depression was assessed using the German version of the BDI-II [45]. The questionnaire consists of 21 items and assesses symptoms experienced in the last two weeks, including hopelessness, irritability, feelings of guilt, and physical symptoms such as fatigue and weight loss. The BDI-II is recommended for use from the age of 13, and its psychometric properties, including convergent validity, are well established in adolescent samples. The internal consistency ranges from a Cronbach's alpha value of .89 to .93, depending on the sample [46]. In our sample, Cronbach's alpha was .94.

#### Beck anxiety inventory (BAI)

The severity of anxiety symptoms over the past 7 days was measured using the German version of the BAI [47]. The BAI consists of 21 items that assess both cognitive and physical aspects of anxiety, such as numbness, tingling, sweating unrelated to heat, and fear of the worst. However, the questionnaire does not measure specific fears. The test has been demonstrated to be suitable for adolescents, and studies have also shown acceptable reliability and validity in adolescent samples. Cronbach's alpha values for clinical samples are .90, while they range from .85 to .90 for nonclinical samples [48]. We calculated a Cronbach's alpha value of .93.

#### Strengths and difficulties questionnaire (SDQ)

The SDQ German version comprises 25 items that assess behavioral strengths and difficulties [49]. These items are categorized into five subscales: 'Emotional Problems', 'Externalizing Behavior Problems', 'Hyperactivity and Attention Problems', 'Peer Problems', and 'Prosocial Behavior'. In addition to the evaluation of the individual subscales, a total problem score (ranging from 0 to 40 points) can be determined from the first four subscales without considering the 'Prosocial Behavior' aspect. This provides information about psychopathological stress. The internal consistency of the total score was measured using Cronbach's alpha, which was found to be .82 [50]. However, in our case, the value was lower at .64.

#### Descriptive statistics of the recorded variables

To get an overview of the study sample’s psychopathological characteristics, Table 2 displays questionnaire data on eating disorder symptoms, depressive symptoms, anxiety symptoms and general psychopathology.

**Table 2:** Description of the recorded variables

	*n*	*M*	*SD*	Clinical cut-off	Above clinical cut-off *n* (%)
FCQ-T-r	88	29.61	13.67	–	–
TFEQ	90	5.74	3.14	–	–
BDI-II	302	27.69	17.83	< 14	214 (70.9%)
BAI	287	19.93	13.63	< 8	223 (77.7%)
SDQ	331	16.49	6.32	< 16	104 (31.4%)

*N* = 342; FCQ-T-r = Food Cravings Questionnaire—reduced (total score); TFEQ = Three-Factor Eating Questionnaire (subscale disinhibition); BDI-II = Beck Depression Inventory (total score); BAI = Beck Anxiety Inventory (total score); SDQ = Strengths and Difficulties Questionnaire (total difficulties score)

#### Anthropometric measurements

Clinic staff recorded the participants' height and weight. Body mass index (BMI) was calculated as weight divided by the square of height (kg/m^2^). The corresponding percentiles were assigned based on the norms of Kromeyer-Hauschild et al. [37].

### Statistical analysis

Prior to statistical analyses, the data were subjected to a comprehensive examination for the presence of missing values. Only fully completed questionnaires were included in the study. The ChEAT total score was found to exhibit a non-normal distribution, which was duly accounted for in the subsequent analyses. The factorial structure and item assignment to subscales were determined using exploratory factor analysis (EFA). For this purpose, principal component analysis with varimax rotation was employed. The model's goodness of fit was assessed using the Kaiser–Meyer–Olkin (KMO) test and Bartlett's sphericity test. Acceptable values were considered to be above .50 for KMO and *p* < .05 for Bartlett's test [51]. The number of factors was determined using Kaiser's criterion and the scree plot. A confirmatory factor analysis (CFA) was performed using weighted least squares mean and variance adjusted (WLSMV) to verify the factor structure. This method is appropriate for non-normally distributed variables and ordinal data [52]. Fit indices, including the chi-squared test, root mean square error of approximation (RMSEA), comparative fit index (CFI), Tucker-Lewis index (TLI), and standardized mean square residual (SRMR), were used to evaluate the model [52–54]. The internal reliability of the ChEAT total score and each subscale was assessed by calculating Cronbach's alpha. The distribution of ChEAT values was analyzed with regard to differences between gender, age, BMI percentiles, and diagnoses of the participants. The Mann–Whitney U-test and the Kruskal–Wallis test were employed to identify any differences in the mean values. As part of the test for convergent and discriminant validity, we correlated the total score of the ChEAT with the FCQ-T-r and TFEQ questionnaires on eating disorders, the BDI-II on depression, and the BAI on anxiety using Spearman rank correlations.

Statistical analyses were conducted using SPSS statistical software (version 29.0.2.0) and the Lavaan package in R (version 4.3.3) for CFA.

## Results

### Descriptive statistics of ChEAT scores

The mean ChEAT score in the study was 13.56 (SD = 13.19), with a range of 0 points to 75 of the maximum possible 78 points. As indicated by the results presented in Table 3, significantly higher scores were observed in females and in participants with a diagnosis of an eating disorder or depressive disorder. Notably, there was no significant variation in ChEAT scores as a function of age or BMI percentile.

**Table 3:** Comparison of ChEAT score by gender, age, BMI percentiles and diagnoses for all participants

	*n*	*M*	SD	*p*
*Gender*				
Girls	197	17.79	14.73	< .001
Boys	145	7.80	7.65	
*Age*				
12–15 years	191	13.52	13.07	.953
16–18 years	151	13.60	13.38	
*BMI percentiles*^*a*^				
Underweight (P3- < P10)	31	14.61	15.81	.251
Normal weight (≥ P10- ≤ P90)	172	12.09	11.93	
Overweight (> P90- ≤ P97)	34	12.85	9.22	
Obesity (> P97)	57	15.25	13.64	
*Diagnoses*^*b*^				
Eating disorder (main or secondary diagnosis)	17	36.76	20.31	< .001
Depressive disorder (unimorbid)	68	16.71	12.15	
Mixed psychiatric	257	11.19	11.10	

*N* = 342; BMI = Body mass index; ^a^BMI-percentiles were based on the norms established by Kromeyer-Hauschild [37]; ^b^Diagnoses were based on the International Classification of Diseases, 10th Revision (ICD-10, World Health Organization [38]), in accordance with the standard guidelines of the German healthcare system

### Exploratory factor analysis

To test the factorial structure of the ChEAT, we conducted an exploratory factor analysis (EFA). The requirements for conducting an EFA were given with a KMO value of .904 and a *p*-value of less than .001 for Bartlett's sphericity test. According to Field , the KMO value for individual items should be greater than .5 [51]. During the testing of this criterion, the items coded inversely were particularly noticeable. However, they were inverted before analysis to ensure that all items had the same coding direction. Item 19, 'I can show self-control around food.', had a borderline score of .545. Item 25, 'I enjoy trying new, rich foods.', scored below the required value with a score of .489. To address this, the EFA was repeated with 24 items, excluding items 19 and 25. The KMO value increased to .908, and Bartlett's sphericity test remained significant at *p* < .001. Based on the eigenvalue criterion (Kaiser's criterion), five factors could be identified. The scree plot revealed two possible solutions: a two-factorial and a five-factorial solution. The five-factor solution was deemed more plausible and aligned better with previously published psychometric analyses. The five-factor scale structure accounted for 63.3% of the total variance, with each factor explaining at least 5% of the variance after rotation. All 24 items loaded .4 or higher on their respective factors. Items 9, 10, and 23 had cross-loadings greater than .4, but these seem plausible in terms of content. These items were assigned according to the strongest loading on the factor. The present five factors contained at least three items and were labeled as 'Body and Weight Concern', 'Dieting', 'Social Pressure', 'Purging and Binge Eating', and 'Food Preoccupation'. Table 4 shows the items and their corresponding factor loadings after EFA.

**Table 4:** Results from Exploratory factorial analysis for 24-item ChEAT

ChEAT item		Factor loading				
		1	2	3	4	5
*Factor 1: Body and Weight Concern*						
11	I think a lot about wanting to be thinner	**.905**	.066	− .004	.152	.100
14	I think a lot about having fat on my body	**.886**	.100	.041	.205	.066
1	I am scared about being overweight	**.830**	.082	.096	.174	.084
12	I think about burning up calories when I exercise	**.618**	.342	− .044	.049	.239
22	I feel uncomfortable after eating sweets	**.591**	.390	.074	.362	.188
10	I feel very guilty after eating	**.514**	.345	.228	**.427**	.234
24	I like my stomach to be empty	**.485**	.371	.123	.255	.340
3	I think about food a lot of the time	**.481**	.292	.342	.184	− .094
*Factor 2: Dieting*						
17	I eat diet foods	.163	**.708**	.207	.139	.078
16	I stay away from foods with sugar in them	.155	**.706**	.316	.048	.247
6	I am aware of the calorie content in foods	.193	**.641**	− .049	.082	.147
7	I try to stay away from foods such as breads	.056	**.613**	.210	.336	.104
23	I have been dieting	**.495**	**.561**	− .007	.157	.112
*Factor 3: Social Pressure*						
8	I feel that others would like me to eat more	.055	.130	**.854**	− .003	.170
13	Other people think I’m too thin	− .130	− .006	**.788**	.078	.095
20	I feel that others pressure me to eat	.200	.221	**.724**	.101	.188
21	I give too much time and thought to food	.359	.351	**.434**	.336	.047
*Factor 4: Purging and Binge Eating*						
26	I have the urge to vomit after eating	.320	.143	− .001	**.767**	.101
4	I have gone on eating binges	.263	.016	.081	**.682**	.052
9	I vomit after I have eaten	.046	**.461**	.012	**.633**	.052
18	I think that food controls my life	.219	.385	.314	**.494**	.139
*Factor 5: Food Preoccupation*						
2	I stay away from eating when I am hungry	.198	.101	.204	0.95	**.758**
5	I cut my food into small pieces	.043	.157	.061	.217	**.718**
15	I take longer than others to eat my meals	.174	.261	.328	− .153	**.549**

*N* = 342. The extraction method was principal component analysis with varimax rotation. Factor loadings above .40 are in bold

### Confirmatory factor analysis

To evaluate the factor structure, a confirmatory factor analysis (CFA) was conducted based on the EFA's results. The previously discarded model with 26 items was compared to the model with 24 items. The 24-item model demonstrated a superior fit to the data with respect to the fit indices, even prior to the implementation of additional adjustments, thereby confirming the rejection of the 26-item model (see Table 5 for factor loadings; for more details of the models, refer to Table 2 in the supplementary information). Some paths were added in the 24-item model between correlated items, as suggested by the modification indices and theoretical explanations. After making the necessary adjustments, the model with 24 items was deemed acceptable based on the following fit indices: *X*^2^/df = 2.04, RMSEA = .055, CFI = .980, TLI = .977, and SRMR = .081.

**Table 5:** Results from Confirmatory factorial analysis for 24-item ChEAT

ChEAT Item		Estimate	Standard Error	Est./SE
*Factor 1: Body and Weight Concern*				
11	I think a lot about wanting to be thinner	.880	.022	38.668
14	I think a lot about having fat on my body	.893	.021	43.095
1	I am scared about being overweight	.892	.021	42.306
12	I think about burning up calories when I exercise	.772	.035	22.158
22	I feel uncomfortable after eating sweets	.908	.021	43.564
10	I feel very guilty after eating	.933	.021	43.612
24	I like my stomach to be empty	.842	.031	27.359
3	I think about food a lot of the time	.648	.050	12.906
*Factor 2: Dieting*				
17	I eat diet foods	.855	.050	17.124
16	I stay away from foods with sugar in them	.926	.049	19.006
6	I am aware of the calorie content in foods	.622	.049	12.581
7	I try to stay away from foods such as breads	.797	.064	12.523
23	I have been dieting	.869	.031	27.927
*Factor 3: Social Pressure*				
8	I feel that others would like me to eat more	.747	.046	16.255
13	Other people think I’m too thin	.409	.077	5.328
20	I feel that others pressure me to eat	1.128	.055	20.487
21	I give too much time and thought to food	.374	.067	5.591
*Factor 4: Purging and Binge Eating*				
26	I have the urge to vomit after eating	.875	.037	23.485
4	I have gone on eating binges	.671	.057	11.831
9	I vomit after I have eaten	.861	.056	15.468
18	I think that food controls my life	.877	.045	19.437
*Factor 5: Food Preoccupation*				
2	I stay away from eating when I am hungry	.788	.051	15.442
5	I cut my food into small pieces	.668	.067	9.905
15	I take longer than others to eat my meals	.719	.060	11.953

*N* = 342; Est. = Estimate; SE = Standard Error

### Internal consistency

Cronbach's alpha of the ChEAT version with 24 items indicated excellent internal consistency with a value of .917. This value was also higher than that of the 26-item version with a Cronbach's alpha of .901. As both items 19 and 25 also showed a negative item-scale correlation, this confirmed our previous exclusion. Factor 1 demonstrated excellent internal consistency, while factors 2, 3, and 4 each exhibited acceptable internal consistency. The internal consistency of factor 5 is the only component that has been identified as questionable. Cronbach's alpha for the individual subscales are presented in Table 6.

**Table 6:** Reliability of the for 24-item ChEAT and its subscales

Dimension	Variance explained	Number of Items	Cronbach’s α
Factor 1: Body and Weight Concern	19.1%	8	.904
Factor 2: Dieting	14.2%	5	.759
Factor 3: Social Pressure	11.3%	4	.769
Factor 4: Purging and Binge Eating	10.8%	4	.740
Factor 5: Food Preoccupation	7.9%	3	.637
ChEAT-total score		24	.917

### Convergent and discriminant validity

As part of the validity test, correlation analyses were conducted between the total score of the ChEAT and its subscales and the total scores of other self-report questionnaires (see Table 7). The ChEAT total score was significantly correlated with the total score of the TFEQ (weak), BDI-II (moderate), and BAI (weak). Only the FCQ-T-r total score did not correlate significantly with the ChEAT total score. The first factor, 'Body and Weight Concern, ' demonstrated a notable correlation with all self-report questionnaires, with the association with the BDI-II being the most pronounced (FCQ-T-r: weak; TFEQ: weak; BDI-II: moderate; BAI: moderate). In contrast, factors 2 'Dieting' and 3 'Social Pressure' demonstrated significant correlations only with the BDI-II (weak) and BAI (weak). Factor 4, 'Purging and Binge Eating, ' demonstrated a significant correlation with all self-report questionnaires, with the strongest correlation observed with the TFEQ (FCQ-T-r: weak; TFEQ: moderate; BDI-II: moderate; BAI: weak). Factor 5, 'Food Preoccupation,' demonstrated only a correlation with the BDI-II (weak) and BAI (weak).

**Table 7:** Correlation between ChEAT-total score and ChEAT-factors and FCQ-T-r, TFEQ, BDI-II and BAI

		ChEAT-total score		Factor 1: Body and Weight Concern		Factor 2: Dieting		Factor 3: Social Pressure		Factor 4: Purging and Binge Eating		Factor 5: Food Preoccupation	
	*n*	*r*	*p*	*r*	*p*	*r*	*p*	*r*	*p*	*r*	*p*	*r*	*p*
FCQ-T-r	88	.107	.151	.172*	.025	.041	.609	− .041	.609	.267**	.001	.013	.872
TFEQ	90	.236**	.002	.287**	< .001	.126	.123	.063	.447	.366**	< .001	− .002	.985
BDI-II	302	.367**	< .001	.440**	< .001	.178**	< .001	.207**	< .001	.319**	< .001	.168**	< .001
BAI	287	.282**	< .001	.341**	< .001	.120**	.007	.150**	< .001	.233**	< .001	.156**	< .001

*N* = 342 (*n* = 50 for each condition); FCQ-T-r = Food Cravings Questionnaire – reduced (total score); TFEQ = Three-Factor Eating Questionnaire (subscale disinhibition); BDI-II = Beck Depression Inventory (total score); BAI = Beck Anxiety Inventory (total score). **p < .01, * *p* < .05

## Discussion

The objective of this study was to assess the reliability and validity of the German version of the ChEAT in a clinical sample. An exploratory factor analysis was conducted to identify the underlying factor structure, followed by a confirmatory factor analysis to confirm the findings. Additionally, the internal consistency of the ChEAT total score and subscales was evaluated. Correlation analyses were conducted with other self-report questionnaires on eating disorders, anxiety, and depression to test convergent and discriminant validity.

The factor analysis identified a five-factor structure in the German version of the ChEAT. The first factor, designated 'Body and Weight Concern', includes items dealing with thoughts about one's own body and the fear of being overweight or gaining weight. The second factor, 'Dieting', pertains to dieting behavior, while the third factor, 'Social pressure', is particularly concerned with the perception that others desire one to eat more and gain weight. The fourth factor, 'Purging and Binge Eating', contains items that cover weight reduction measures (especially vomiting), but also the feeling of loss of control in the context of binge eating. The last factor, designated 'Food Preoccupation', pertains to eating behavior, for instance the duration of food intake. The number of factors corresponded to the results of studies from Spain, Japan, and Belarus [27, 28, 30]. However, in terms of content, the Spanish study demonstrated the greatest overlap with the German version. The content focus of the five factors matched, and the first three factors exhibited notable similarities in the assignment of the items. For instance, items 1, 11, 12, 14, and 25 of the factor 'Preoccupation with thinness' were found to be congruent, with the factor 'Dieting' being assigned the common items 6, 7, 16, and 17. Additionally, the factor 'Social Pressure' was found to be congruent in items 8, 13, and 21. Although the Spanish sample was considerably larger, the comparable structure may possibly be attributed to the similar average age of the participants. In the majority of studies, the average age was between 10 and 12 years, whereas in our sample the average age was 15.10 years, which is comparable to the average age of 13.84 years in the Spanish study [27].

Upon examination of the underlying assumptions of a factor analysis, it became evident that the inversely coded items 19 and 25 were particularly prominent. This observation persisted throughout subsequent analyses, which is why we ultimately excluded these items and proceeded with the model comprising only 24 items. Previous studies have recommended the exclusion of one or both items [18, 26, 28, 31, 39]. The researchers from Finland posited that the inverse-coded items were not sufficiently clear to the participants, resulting in response bias. This was attributed to the tendency of participants to answer the items in a consistent direction [31]. Furthermore, we classified items 19 and 25 as problematic due to the wording employed in their formulation. In our estimation, the aspects 'self-control in relation to food' and 'rich foods' are not sufficiently delineated for children and adolescents, which may result in response bias.

The reliability test using Cronbach's alpha demonstrated excellent internal consistency for the German version of the ChEAT, which contains 24 items. The value of .917 is notably higher than that observed in comparable international studies. The five subscales also demonstrated acceptable internal consistency, with the exception of the factor 'Food preoccupation', which exhibited a relatively low value of .637. The low Cronbach's alpha value may be attributed to the fact that the subscale comprises a mere three items. It is important to note that the reliability coefficient of a scale is contingent upon the number of items included on the scale [55]. However, the items are clinically relevant and do not fit sufficiently to another factor. Accordingly, we decided to retain the subscale, particularly in view of the fact that significantly low Cronbach's alpha values for individual subscales have been tolerated in other studies [27, 28].

Validity was evaluated by testing convergent and discriminant validity. The results indicated that a significant weak correlation was observed between the ChEAT total score and the TFEQ, while no significant correlation was found between the ChEAT total score and the FCQ-T-r. With respect to the subscales of the ChEAT, the FCQ-T-r and the TFEQ exhibited weak and in part moderate significant correlations with factors 1 'Body and Weight Concern' and 4 'Purging and Binge Eating'. The correlations of the TFEQ were stronger in all cases. Upon further examination, however, this result of the correlation analysis appears to be a reasonable conclusion. The TFEQ, or more specifically the selected subscale 'disinhibition', exhibits a greater degree of overlap with the ChEAT in terms of content. Both tools assess eating behavior in general and, as a result, are not limited to a specific eating disorder. Therefore, the items of the TFEQ encompass aspects corresponding to nearly all subscales of the German version of the ChEAT. For example, the 'Dieting' factor of the ChEAT can be assigned the item 'I have gone on reducing diets more than once.', while the 'Purging and Binge Eating' factor can be described by the items 'Sometimes things just taste so good that I keep on eating even when I am no longer hungry.' or 'Sometimes when I start eating, I just can't seem to stop.' The item 'Without even thinking about it, I take a long time to eat' can be assigned to the 'Food preoccupation' factor. In contrast, the FCQ-T-r is exclusively focused on food craving, thoughts about eating, and loss of control, which most closely aligns with binge eating disorder. Consequently, the FCQ-T-r assesses a distinct construct in comparison to the ChEAT and the TFEQ. Additionally, a relatively limited number of participants (*n* = 88) responded to the FCQ-T-r, which could have influenced the results. Subsequently, correlation analyses were conducted between the ChEAT total score and the questionnaires on depression (BDI-II) and anxiety (BAI) as part of the investigation of discriminant validity. Both questionnaires demonstrated a statistically significant weak or moderate correlation with the ChEAT total score and all factors. These findings are consistent with those of previous analyses conducted in the adult sector, in which the EAT-26 was correlated with the BDI and BAI [40, 56, 57]. This result supports the findings of the aforementioned studies on the comorbidity of depression and anxiety disorders in patients with eating disorders.

The average ChEAT score was 13.56 points, which was higher than in previous studies. Only one study from the USA found even higher average scores, but it only recruited female participants from a middle school, which could be the reason for the higher scores [18]. In the current study, high scores can probably be attributed to the mixed psychiatric sample and the fact that participants were in acute inpatient treatment at the time of recruitment. In addition to participants with eating disorders, our sample included more than one-third of participants who received a primary diagnosis of depressive disorder. These depressive patients exhibited higher mean scores on the ChEAT than participants with other psychiatric disorders. The symptoms of this disorder can manifest themselves through changes in appetite, but also weight gain or weight loss [58]. These aspects are also recorded via the items of the ChEAT, for example through statements such as 'I stay away from eating when I am hungry' or 'I take longer than others to eat my meals'. Depression and eating disorders frequently co-occur, with numerous studies indicating that major depressive disorder is the most prevalent comorbidity in individuals with eating disorders, and that depressive symptoms tend to increase with the severity of the eating disorder [10, 15, 16, 59, 60]. A study of women with major depressive disorder or anxiety disorders, for example, demonstrated that the prevalence of eating disorders is four times higher in these illnesses [61].

We also compared the average ChEAT score within different groups. This showed a statistically significantly higher average score for female participants and those with a diagnosed eating disorder. There are a number of risk factors for developing an eating disorder, and female gender is one of them, consistent with our findings [20, 62]. For example, one study shows that girls are seven times more likely to develop pathological body dissatisfaction, which is considered a predisposing factor for eating disorders [11].

### Strengths and limits

In this section, we will address the limitations of this study, which include incomplete data on BMI for participants. While conclusions could be drawn regarding the relationship between ChEAT scores and BMI percentiles, the incomplete data on weight limits the generalizability of the findings. Furthermore, some data was gathered through self-assessment questionnaires, which may have introduced bias and constrained some interpretations. Some of the measures utilized in this study were not exclusively designed for children or adolescents, which may restrict the generalizability of our findings to younger populations. Our study design was a cross-sectional study. Consequently, the findings of this study can be evaluated as a snapshot. A longitudinal study would be a logical next step. A further limitation is the relatively small number of participants for a validation study, particularly for those patients with an eating disorder. Likewise, a greater number of participants with a diagnosed eating disorder should be included, as in the current study, due to the low proportion of participants with this disorder, no meaningful cut-off determination was possible. With regard to the factor analysis, it is important to note that the identical data set was used for the CFA and EFA, which may introduce potential limitations to the study. While this issue may diminish with an augmented sample size, it can result in the overfitting of the model and, consequently, a distortion of the CFA results. In subsequent studies examining the German factor structure of the ChEAT, it is recommended that the CFA and EFA be conducted in distinct samples.

A notable strength of this study is the diverse psychiatric composition of the sample, which has not yet been investigated in this form. By employing the questionnaire in a clinical setting, conclusions can be drawn regarding its suitability as a screening tool for detecting eating disorders in general, as well as for identifying comorbid disorders in the context of other underlying illnesses. It is also noteworthy that the internal consistency of the total score of the ChEAT was exceptionally high, particularly when compared to other international studies.

## Conclusion

In summary, the results from our sample suggest that the German version of the ChEAT is a valid instrument for measuring eating disorder symptoms. However, further studies with larger samples and a broader range of healthy control groups and participants with eating disorders are needed to confirm the factor structure of the German version. Convergent validity should be re-evaluated using a validated and comprehensive eating disorder questionnaire that is suitable for children and adolescents.

### What is already known on this subject?

Eating disorders are prevalent worldwide, with origins potentially emerging in early childhood and reaching a peak during adolescence. Therefore, it is of great importance to detect eating disorder symptoms at an early stage to provide appropriate treatment. The ChEAT is an internationally validated tool for detecting eating disorder symptoms in children. However, validity studies in various countries have shown considerable differences in its factor structure and number of items.

### What this study adds?

In the absence of validity studies from German-speaking countries, the good psychometric properties of the German version of the ChEAT were confirmed in an adolescent clinical sample. The use of the ChEAT in a mixed psychiatric sample demonstrated that increased average scores can occur not only in female patients and patients with eating disorders but also in those with depressive disorder.

## Supplementary Information

Below is the link to the electronic supplementary material.

Supplementary file 1 (PDF 297 KB)

## Data Availability

No datasets were generated or analysed during the current study.
