# Supplementary material for: Children's eating attitudes test (ChEAT): reliability and validation in German children and adolescents based on clinical data
Source: Eat Weight Disord. 2025 Aug 14;30(1):64. doi: 10.1007/s40519-025-01773-w (PMC12354608; doi:10.1007/s40519-025-01773-w)
Supplement: Supplementary file 1 — (PDF 297 KB) [file 40519_2025_1773_MOESM1_ESM.pdf]

Supplementary information

Eating and Weight Disorders - Studies on Anorexia, Bulimia and Obesity

Children's Eating Attitudes Test (ChEAT): Reliability and Validation in German Children and Adolescents Based on Clinical Data

Lena Nonnast[1], Laura Maria Derks[1] (0000-0001-5333-0618), Natalie Deux[1], Martin Holtmann[1] (0000-0003-2644-6601), Tanja Legenbauer[1] (0000-0002-1580-9991)
[1] LWL University Hospital Hamm for Child and Adolescent Psychiatry, Psychotherapy and Psychosomatic, Ruhr-University Bochum, Hamm, Germany

Correspondence: Tanja Legenbauer (tanja.legenbauer@lwl.org)

Table 1: Overview of international validity studies of the ChEAT *(no claim to completeness or correctness)*

**Country**	**Sample**	**ChEAT-mean score**	**Cronbach's α**		**Factorstructure**			
			total score	subscale	number	factor name	items	confirmatory FA
**Turkey** Bozkurt et al. (2023)	size: 331<br>participants: school children<br>age range: 8-15 years<br>mean age: 11.73<br>gender ratio: 59% girls	12<br>girls: 13<br>boys: 11	0.75<br>(26 items)	I: 0.67<br>II: 0.63<br>III: 0.71	3	I: Preoccupation with thinness and food<br>II: Social pressure to eat<br>III: Dieting	I: 1, 3, 4, 10, 11, 14, 18, 21, 24, 25 (10)<br>II: 8, 13, 15, 20 (4)<br>III: 2, 5, 6, 7, 9, 12, 16, 17, 19, 22, 23, 26 (12)	missing
**Portugal** Teixera et al. (2012)	size: 956<br>participants: school children<br>age range: 11-18<br>mean age: 15.78<br>gender ratio: 59% girls	8.61<br>girls: 9.44<br>boys: 7.28	0.73<br>(24 items)	I: 0.79<br>II: 0.56<br>III: 0.51<br>IV: 0.71	4	I: Fear of getting fat<br>II: Restrictive and purging behaviours<br>III: Food preoccupation<br>IV: Social pressure to eat	I: 11, 14, 1, 12, 22, 23, 10, 2, 24, 25 (10)<br>II: 7, 16, 17, 6, 9, 5 (6)<br>III: 3, 21, 4, 18, 19 (5)<br>IV: 20, 8, 13 (3)	missing
**USA** Ranzenhofer et al. (2008)	size: 417<br>participants: 220 overweight patients and control group<br>age range: 6-18<br>mean age: 10.8<br>gender ratio: 54% girls	8.55<br>girls: 9.6<br>boys: 7.5	0.78<br>(16 items)	I: 0.74<br>II: 0.66<br>III: 0.55<br>IV: 0.52	4	I: Body/weight concern<br>II: Food preoccupation<br>III: Dieting<br>IV: Eating concern	I: 1, 11, 12, 14 (4)<br>II: 3, 4, 21 (3)<br>III: 5, 6, 7, 17, 23 (5)<br>IV: 2, 8, 15, 20 (4)	missing
**USA** Smolak et al.	size: 308<br>participants: school children	15.74	0.87<br>(26 items)		4	I: Dieting<br>II: Restricting and purging	I: 11, 12, 14, 1, 23, 10, 6, 22, 16 (9)	missing

(1994)	age range: missing<br>mean age: 13.2<br>gender ratio: only girls		0.88<br>(25 items)<br><br>0.89<br>(23 items)			III: Food preoccupation<br>IV: Oral control	II: 17, 9, 26, 24, 2, 7 (6)<br>III: 21, 3, 18, 4 (4)<br>IV: 8, 13, 20, 15 (4)	
**China**<br>Huang et al.<br>(2022)	size: 906<br>participants: school children<br>age range: 8-16<br>mean age: 10.55<br>gender ratio: 47% girls	9.21<br>girls: 9.7<br>boys: 8.79	0.819<br>(26 items)	I: 0.73<br>II: 0.70<br>III: 0.65<br>IV: 0.54	4	I: Fear of getting fat<br>II: Dieting and purging behaviors<br>III: Bulimia and food preoccupation<br>IV: Social pressure to eat	I: 11, 1, 14, 12, 17, 16, 6 (7)<br>II: 26, 9, 10, 2, 24, 7, 23, 22, 19 (9)<br>III: 4, 3, 21, 25, 18, 13 (6)<br>IV: 13, 8, 20, 15, 5 (5)	χ2/df = 2.943<br>RMSEA = 0.046<br>CFI = 0.883<br>TLI = 0.869
**Finland**<br>Lommi et al.<br>(2020)	size: 339<br>participants: school children<br>age range: 10-15<br>mean age: 11.9<br>gender ratio: 55% girls	2.55 (26)<br>2.03 (24)	0.79<br>(26 items)<br><br>0.84<br>(24 items)	missing	4	I: Concerns about weight<br>II: Limiting food intake<br>III: Pressure to eat<br>IV: Concerns about food	I: 1, 6, 7, 10, 11, 12, 13, 14, 17, 18, 22, 23, 24 (13)<br>II: 2, 4, 5, 9, 26 (5)<br>III: 8, 15, 20, 21 (4)<br>IV: 3, 16 (2)	*26 items*<br>CFI = 0,872<br>RMSEA = 0.028<br>SRMR = 0.101<br>GFI = 0.976<br>Chi-square 0.003<br><br>*24 items*<br>CFI = 0,959<br>RMSEA = 0.017<br>SRMR = 0.091<br>GFI = 0.971<br>Chi-square 0.144
**Belgium**<br>Theuwis et al.<br>(2009)	size: 166<br>participants: 65 overweight patients and control group<br>age range: 8-15<br>mean age: 11.41<br>gender ratio: 62.5% girls	missing	0.85<br>(26 items)<br><br>0.88<br>(24 items)	missing	4	I: Dieting attitudes and feelings of social pressure to eat<br>II: Concerns about weight and body shape<br>III: Eating concerns<br>IV: Food preoccupation and keeping control	I: 7, 8, 9, 10, 13, 16, 17, 18, 20, 21, 22, 24, and 26 (13)<br>II: 1, 11, 12, 14 (4)<br>III: 2, 6, 17, 23, 24 (5)<br>IV: 3, 4, 15, 19, 21(5)	missing
**Spain**<br>Sancho et al.<br>(2005)	size: 1336<br>participants: school children<br>age range: 9-13<br>mean age: 11.37	8.95<br>girls: 9.01<br>boys: 8.88	0.71<br>(20 items)		4	I: Fear and preoccupation of getting fat<br>II: Social pressure to eat<br>II: Food preoccupation	I: 11, 14 ,1 ,10 ,23 ,12 ,2 (7)<br>II: 8, 20, 13, 15 (4)<br>II: 3, 4, 21 (3)	missing

	gender ratio: 48.7% girls					IV: Food restriction	IV: 16, 22, 6, 23, 17, 7 (6)	
**Canada** Legendre et al. (2021)	size: 1092<br>participants: school children<br>age range: 8-12<br>mean age: 10.31<br>gender ratio: 56.9% girls	1.92<br>girls: 2.24<br>boys: 1.49	0.86<br>(12 items)	I: 0.69<br>II: 0.95<br>III: 0.86<br>IV: 0.92	4	I: Dieting<br>II: Weight preoccupation<br>III: Food preoccupation<br>IV: Social pressure<br><br>CFA $\chi2/df$)=2.10; CFI=0.987; RMSEA=0.033, 90% CI (0.024 to 0.042)	missing	χ2/df = 2.10<br>CFI=0.987<br>RMSEA=0.033
**Croatia** Ambrosi-Randic et al. (2005)	size: 225<br>participants: school children<br>age range: 10-15<br>mean age: 12.5<br>gender ratio: only girls	10.71	0.71<br>(25 items)		4	I: Dieting<br>II: weight and food preoccupation<br>III: oral contral<br>IV: mixed	I: 23, 22, 17, 16, 6, 24 (6)<br>II: 14, 1, 10, 11, 4, 18, 19, 25 (8)<br>III: 8, 13, 20, 15, 9 (5)<br>IV: 21, 2, 3, 26, 7, 12 (6)	missing
**Spain** Rojo-Moreno et al. (2011)	size: 38504<br>participants: school children<br>age range: 13-17<br>mean age: 13.84<br>gender ratio: 49.7% girls<br><br>*sample II*<br>size: 968<br>participants: 49 ED patients and control group<br>age range: 13-17<br>mean age: 14.1<br>gender ratio: 49.8%	7.55<br>girls: 9.03<br>boys: 6.09	0.86<br>(26 items)	I: 0.84<br>II: 0.66<br>III: 0.66<br>IV: 0.64<br>V: 0.58	5	I: Preoccupation with thinness<br>II: Dieting<br>III: Social pressure<br>IV: Purging<br>V: Preoccupation with food and oral control	I: 11, 14, 1, 12, 24 (5)<br>II: 16,17,7, 23, 6, 2, 5 (7)<br>III: 8, 20, 13, 15 (4)<br>IV: 26, 9, 10, 22 (4)<br>V: 3, 4, 21, 25, 19, 18 (6)	RMSEA = 0.058
**Japan** Chiba et al. (2016)	size: 7120<br>participants: 44 AN patients and control group<br>age range: 10-15<br>mean age: missing<br>gender ratio: 50.3% girls	6.9<br>girls: 7.94<br>boys: 5.86	0.79<br>(26 items)<br><br>0.81<br>(25 items)	I: 0.82<br>II: 0.39<br>III: 0.58<br>IV: 0.62<br>V: 0.59<br><br>25 items<br>I: 0.82	5	I: Preoccupation with thinness<br>II: Food preoccupation<br>III: Dieting<br>IV: Social pressure to eat<br>V: Purging	I: 1, 10, 11, 14, 23 (5)<br>II: 3, 4, 18, 21, 24, 25 (6)<br>III: 2, 5, 6, 7, 12, 16, 17, 19, 22 (9)<br>IV: 8, 13, 15, 20 (4)<br>V: 9, 26 (2)	RMSEA = 0.050

				II: 0.61<br>III: 0.58<br>IV: 0.62<br>V: 0.59				
**Poland**<br>Pilecki et al. (2013)	size: 375<br>participants: school children<br>age range: missing<br>mean age: 11.8<br>gender ratio: 53.7% girls	7.6<br>girls: 7.65<br>boys: 7.55	missing	I: 0.84<br>II: 0.78<br>III: 0.70<br>IV: 0.50<br>V: 0.47<br>VI: 0.34<br>VII: 0.002	7	I: Desire to slim<br>II: Pressure to gain weight<br>III: Compulsive-bulimic<br>IV: Diet-weight loss<br>V: Excessively healthy eating<br>VI: Pre-compensatory-bulimic<br>VII: Pleasure in overeating	I: 11, 1, 14, 12, 2 (5)<br>II: 15, 13, 8, 20 (4)<br>III: 4, 3, 21 (3)<br>IV: 16, 7, 23, 22 (4)<br>V: 5, 6, 18, 17, 19 (5)<br>VI: 10, 26 (2)<br>VII: 24, 25 (2)	missing
**Sweden**<br>Edlund et al. (1994)	size: 197<br>participants: school children<br>age range: 11-16<br>mean age: 13.3<br>gender ratio: 49.3% girls	missing<br>girls: 3.93<br>boys: 2.65	missing	missing	missing	missing	missing	missing

Table 2: Comparison between the 24-item and 26-item model

	24-item model	26-item model
exploratory factor analysis		
Kaiser-Meyer-Olkin test	**.908**	.904
Bartlett's sphericity test	<.001	<.001
confirmatory factor analysis<br>*(before further adjustments in accordance with modification indices)*		
X^2/df<br>< 2 good fit<br>< 3 acceptable fit	**3.22**	3.61
CFI > .95	**.950**	.949
TLI > .95	**.944**	.942
RMSEA < .06	**.081**	.088
SRMR < .8	.110	.110
internal reliability		
Cronbach's alpha	**.917**	.901
